# Determination of virulence determinants of *Escherichia coli* strains isolated from patients with colorectal cancer compared to the healthy subjects 

**Published:** 2019

**Authors:** Omid Zarei, Mohammad Reza Arabestan, Amir Majlesi, Younes Mohammadi, Mohammad Yousef Alikhani

**Affiliations:** 1 *Department of Microbiology, Faculty of Medicine, Hamadan University of Medical Sciences, Hamadan, Iran*; 2 *Department of Gastroenterology, Faculty of Medicine, Hamadan University of Medical Sciences, Hamadan, Iran*; 3 *Modeling of Non-communicable Diseases Research Center, Department of Epidemiology, School of Public Health, Hamadan University of Medical Sciences, Hamadan, Iran*; 4 *Brucellosis Research Center, Hamadan University of Medical Sciences, Hamadan, Iran *

**Keywords:** E. coli; Malignancy; Virulence factor

## Abstract

**Aim:**

This study determined the genes encoding the binding and receiving factors of iron and microbial biofilm in *E. coli* strains isolated from mucosal samples of patients with colorectal cancer and inflammation of the colorectal compared to healthy people.

**Background:**

Colorectal cancer is one of the most important malignancies in recent years. *Escherichia coli* is the most important infectious agents associated with colorectal cancer that has numerous virulence factors such as iron uptake and adhesion factors included in the process of inflammation and colorectal cancer.

**Methods:**

Of the three healthy, inflammatory bowel diseases and colorectal cancer groups, 40 *Escherichia coli* strains isolated after confirmation by biochemical and molecular methods. After determining the isolates phylogroups, the frequency of genes was measured by PCR method. The biofilm formation of isolates was performed using Crystal Violet method.

**Results:**

In the determination of the bacteria phylogroups, the colorectal cancer isolates had a maximum incidence of phylogroups B2 and A. In the analysis of *fimH*,* papA*,* papC*,* iutA*,* ireA *and* fyuA* *genes, *the highest frequency was observed in these two phylogroups. The presence of *ireA* gene in bacterial isolates from three groups showed a significant difference (*P* value: 0.004). There was also no significant difference in biofilm productions in *Escherichia coli* strains isolated from the three groups.

**Conclusion:**

Unlike previous studies focusing solely on *Escherichia coli* toxins, we found that iron absorption and adhesion factors could be effective in developing inflammatory bowel diseases and colorectal cancer. It was also determined that biofilm formation is a specific characteristic of *Escherichia coli* isolated from the healthy colon.

## Introduction

 Colorectal cancer (CRC) is a standout amongst the numerous frequently distinguished malignancies and the fourth fundamental reason for malignancy mortality in the world (1). CRC is a heterogeneous disease and genetic mutations, epigenetic changes, lifestyles, and infectious agents are the starting point for cancer processes (2). Approximately 20% of the reasons for CRC is related to the infectious causes. Different bacteria are the causative agents of inflammatory bowel diseases (IBD) and CRC (3). Many comments have been made on the role of intestinal bacteria as pathogens in IBD in kids and adults (4). In the human intestinal tract 10^13^-10^14^ bacteria, called microbiota, which are essential for homeostasis (5). The collapse of this microbial balance has been watched in CRC patients (6,7). Although *Escherichia coli* (*E. coli)* is a symbiosis in the human intestine, several studies have shown the association between the binding of this bacterium to the intestinal mucosa and its role in the incidence of CRC (8). The result of the defect in protective mucus layer increases the probability of binding between bacteria and epithelial cells, which is the first step in biologic changes, inflammation, and eventually CRC (9). *E. coli* connects to the intestinal mucosa more often in the ileum and colon area, and there are reports that there is an association between increased *E. coli *mucosal connective tissue and colorectal cancer (10). Following the acquisition of different virulence factors, *E. coli* can be classified in eight phylogroups (A, B1, B2, C, D, E, F, and clade I), using the technique developed by Clermont *et al*. (11). Worldwide phylogenetic analyses have demonstrated that strains belonging to phylogroup B2 and to a lesser extent to phylogroup D, carry some virulence factors which are responsible for a variety of intestinal and extra intestinal diseases (12). The adhesion is the first stage in colonization and infectious disease, which is facilitated by fimbriae agents (13). In IBD such as Crohn's disease, the amount of expression glycoprotein CEACAM6 as a receptor on the surface of intestine epithelial cells for type 1 pili (*fimH*) of *E. coli* have been increased (14). In addition, the P fimbrial may be of particular importance. The most important subunits are *papC* and *papA*, which are located in the outer membrane of the bacteria (15). Once connected, bacteria create biofilms under certain conditions, such as environmental stresses. In the event of biofilms attacking the mucous membrane of the intestine and contact with the epithelial cells of the intestine, a pathological situation is created (16). Over and above binding, iron absorption factors also affected the pathogenicity of *E. coli *(17). There are relatively few studies on the association between *E. coli* iron uptake and adhesion factors and colorectal cancer. Therefore, the purpose of this study was to determine the genes encoding the binding and receiving factors of iron and microbial biofilm in *E. coli *strains isolated from mucosal samples of patients with colorectal cancer and inflammation of the colorectal in comparison with healthy people. 

## Methods

**Patients and biopsy specimens**

One hundred and forty-two patients included in this study were hospitalized in the educational hospitals of Hamadan University of Medical Science, Hamadan, west of Iran, from September 2015 until February 2017. The patients and control groups included 54 men and 66 women (Table 1), with a mean age of 56 years (age range 16 – 81 years) and mean disease duration of 5-13 years provided a signed agreement for this study, and the protocol was approved by the local ethics committee of the Hamadan University of Medical Science (IR.UMSHA.REC.1395.298).

**Table 1:** The number of males and females were sampled in each group

**Patient groups**		**Sex**	
		**Male NO (%)**	**Female NO (%)**
Type	Normal	20 (50.0)	20 (50.0)
	IBD	19 (47)	21 (52.5)
	Cancer	17 (42.5)	23 (57.5)
Total		56 (46.7)	64 (53.3)

Intestinal biopsies were obtained from terminal ileum and the colon in 40 patients with CRC and 40 patients with IBD. In addition, from the ileum and colon of 40 individuals who had no significant pathological findings following endoscopic examination for changes in stool habits, abdominal pain, upper gastrointestinal bleeding or cancer and were considered as controls (18). During a colonoscopy, two biopsy samples were taken from each person for routine pathology assessment and bacteriological study. Specimens were collected in 2-ml screw-cap vials filled with 0.85 ml of brain heart infusion broth (Oxoid, Cambridge, United Kingdom) and 0.15 ml of glycerol (Sigma-Aldrich, St. Louis, MO) and immediately stored at **-**70° C.

**Table 2:** Related results virulence factors and three groups studied

** Virulence Factors**	**Normal no (%) **	**IBD no (%)**	**Cancer no (%) **	**P- value**
*fimH*	29 (72.5)	30 (75)	33 (82.5)	0.546
*papA*	15 (37.5)	15 (37.5)	12 (30)	0.719
*papC*	10 (25)	16 (40)	9 (22.5)	0.176
*iutA*	16 (40)	24 (80)	21 (52.5)	0.195
*fyuA*	19 (47.5)	23 (57.5)	17 (42.5)	0.393
*ireA*	6 (15)	20 (50)	14 (35)	0.004*

* P- value <0.05: significant

**Table 3:** Frequency of virulence factors in each Phylogroups

**Phylogroups**	**Virulence Factors** **No (%)**				**p-Value**
	**D**	**B** **2**	**B** **1**	**A**	
*fimH*	36 (39.1)	12 (13.0)	35 (38.0)	9 (9.8)	0.447
*papA*	15 (35.7)	9 (21.4)	13 (31.0)	5 (11.9)	0.722
*papC*	13 (37.1)	8 (22.9)	9 (25.7)	5 (14.3)	0.852
*fyuA*	23 (39)	6 (10.2)	22 (37.3)	8 (13.6)	0.281
*iutA*	25 (41.0)	7(11.5)	23 (37.7)	6 (9.8)	0.579
*ireA*	15 (37.5)	1 (2.5)	21(52.5)	3 (7.5)	0.276

* P- value <0.05: significant

**Table 4:** The relationship between biofilm formations in strains isolated from the three groups studied

**Biofilm**	**Normal no (%)**	**IBD no (%)**	**Cancer no (%)**	**P - value**
without	6 (15)	12 (30)	11 (27.5)	0.569
Weak	22 (55)	19 (47)	21 (52.5)	
Mediate	11 (27.5)	8 (20)	8 (20)	
Strong	1 (2.5)	1 (2.5)	0 (0.0)	

* P- value <0.05: significant

**Treatment of biopsy specimens and bacterial culture**

For *E. coli* isolation, biopsy specimens (15 mg each) from CRC, IBD, and control patients were first washed in 500µl of physiologic saline with 0.016% dithioerythritol to remove the mucus and then shaken 3 times in 500µl of physiological saline for 30s. After a fourth wash, the biopsy specimens were hypotonically lysed by vortexing for 30 min in 500 µl distilled water (19). One hundred microliters of the cell debris left after hypotonic lysis was plated in 10-fold dilution steps onto MacConkey agar. After 24h of incubation at 37° C, all bacterial colonies were isolated and subcultured in nutrient agar and successively identified by the biochemical tests (20), and confirmed by detection of *rpoB *gene (21)*. E. coli* strains from controls were retrieved only from descending colon biopsy specimens.

**DNA Extraction and Amplification**

All isolates were prepared by inoculating a single colony into 1 ml of Luria Bertani (LB) broth and incubated at 37° C with shaking (100 rpm) overnight. DNA was extracted using boiled lysates and collected at 20° C until used (22, 23). Several rounds of PCR were performed to detect *E. coli* genes as the following procedure: In this study, specific primers of *fimH* (24), *papA, papC, iutA, fyuA* (25), and ir*e*A (26) genes were used. One microliters of the extracted DNA was included in 15μl of PCR master mix, 1μl of each primers (10 pmol), and 7μl of ddH2O. Likewise, pure water was used as negative control for all rounds of PCR.

Cycling program for rpoB gene amplification was as follows: an initial denaturation at 94° C for 5 min and then 35 cycles consisting of 94° C for the 60s, 57° C for 50s, and 72° C for 60s followed by a final extension at 72° C for 10 min. Cycling program for adhesion, iron uptake and phylogruoping encoding genes were the same with different annealing temperature. A 10µL of each PCR products was subjected to electrophoresis on a 2% w/v agarose gel, followed by staining with 1µl Gel Red 20× (Biotium®) and by 125 volts for 45 minutes and analyzed by Gel Doc Transilluminator system (VilberLourmat model).

**Figure 1:**
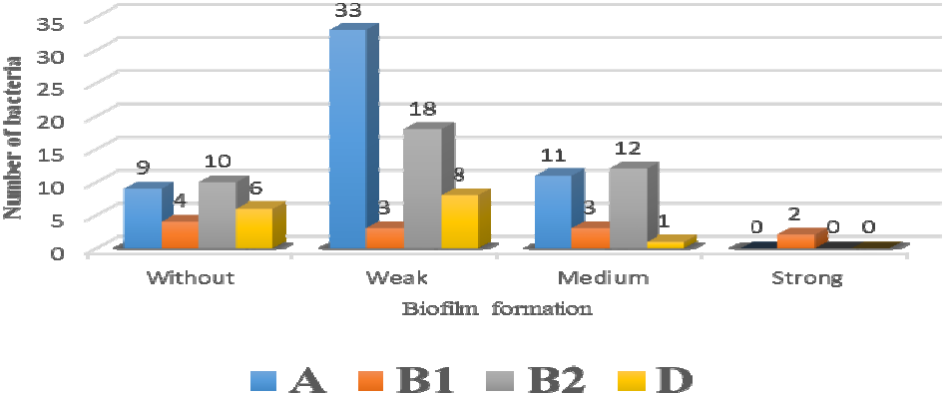
Relationship between phylogroups and biofilm formation

**Biofilm assay**

Biofilm production by microtiter plate method (MTP) performed as follows: isolates of *E. coli* were cultured overnight in Trypticase Soy Broth (TSB) (Oxoid, UK) supplemented with 1% glucose (TSBglu). A volume of 200µl was transferred to wells of the sterile 96 wells microtiter polystyrene tissue culture plate (Becton Dickinson, Franklin Lakes, NJ, USA). Each isolate was tested twice. After cultivation for 24 hr. at 37° C, the contents of the wells were discarded and the wells were gently washed three times with 200 µl sterile phosphate-buffered saline PBS (pH 7.2). The dye bound to adherent bacteria was solubilized with 200 µL of 95% (v/v) ethanol per well. Followed by 200 µl crystal violet (0.1%) for 30 min. at room temperature for biofilm staining, followed by washing 3 times with tap water. The absorbance (OD) of the remaining surface-adsorbed cells of the individual wells was read on a spectrophotometer (ELX 800 Universal Microplate Reader Bio-TEC Instruments, INC) at 595nm. *E. coli* DH5-alpha and non-bacterial culture medium were used as a positive and negative control, respectively. The following values were assigned for biofilm determination: Non-biofilm producer: OD595 ≤1, Weak biofilm producer: 1< OD595 ≤ 2, Medium biofilm producer: 2<OD595 ≤ 3 and Strong biofilm producer: OD595 >3 (27).

**Statistical Analysis**

Chi-square, Fisher's Exact Test, McNemar's test and analysis of variance (ANOVA) tests were used to assess difference among study groups and P-value less than 0.05 was considered statistically significant. The statistical analyses were performed using SPSS version 11. 

## Results

In this study *E. coli* were isolated from biopsy samples of IBD (CD and UC) 78% (n = 40/51); control 93%, (n = 40/43); and cancer 83% (n = 40/48) groups. Among the phylogroups obtained, groups B2 and A were the most frequent in IBD (52.5%, 25%) and cancer groups (30%, 52.5%), respectively. In the case of the *fimH* gene, its frequency in control subjects was lower than that of the other two groups. In such cases, the frequency of control (72.5%) was 40.29%, in inflammatory patients it was 75% (40.30%) and cancer (82.5%) (40.33%). The distribution of *papA* and *papC* genes was not significantly different between the three groups. The frequency of these genes was higher in the control subjects than in the cancer patients. However, comparing these genes between healthy and inflammatory individuals about *papA* gene in equilibrium or *papC* gene was slightly higher in inflammatory individuals. In the three genes involved in the of iron uptake *iutA*, *fyuA*, and *ireA*, IBD patients had the highest frequency of 80%, 57.5%, and 50%, respectively and only *ireA* gene had a significant difference between the three groups (0.004). The results of the frequency of genes are shown in Table 2. In the analysis carried out the frequency of virulence factors in each of the four phylogroups, maximum frequency belonged to the phylogroups B2 and A, and the results of this analysis can be found in Table 3. The biofilm formation in three weak, moderate and strong categories was higher in strains isolated from normal people and in general, no significant difference was found between their frequencies (Table 4). Also, in the checking of the relationship between biofilms and phylogroups, as shown in chart 1, we found that only two strains of Phylogroup B1 were able to produce strong biofilms from all four phylogroups. There was a statistically significant difference between the amount of biofilm and phylogroup formation for B2 and A. Thus, the average biofilm formation was in the strains belonging to the phylogroups B2 and A 12(44.4%) and 11(40.7%), respectively.

## Discussion

Colorectal cancer is one of the major widespread forms of cancer and IBD included ulcerative colitis (UC) and Crohn's disease (CD) have an amplified risks of CRC (28, 29). It has been recommended that the role of *E. coli* in CRC promotion and progress is associated to chronic inflammation, which can result from bacterial infection via its effects on both the host and the microbiota (30). In our study, *E. coli* were isolated from biopsy samples of IBD, control and cancer groups between 78% and 93%. In parallel, different studies have reported that between 71% and 82% of patients with colonic carcinoma are highly colonized by *E. coli* compared to controls (31). Among the phylogroups mentioned for *E. coli,* a greater relative abundance belonging to the B2 and D phylogenetic group has been reported in CRC and IBD patients (6, 32). We found surprisingly different phylogenetic distribution from preceding studies, including a lower prevalence of group D, *E. coli *was isolated from cancer and IBD samples, but the frequency of phylogroups B2 and A was abundant in these samples. This probably can be a confirmation that IBD can be one of the causes of CRC and approve the results of previous researches and contrary to some studies showing that the distribution of phylogroup A among the controls and cancer patients is not significantly different (33). In most studies conducted on isolated *E. coli* strains of colorectal cancer patients, they have investigated pks-related genes and confirmed their association with colorectal cancer (34, 35). Almost all bacteria causing intestinal infections adheres to the gut mucosa. 

This property enables the bacteria to colonize the gut and defend against mechanical exclusion from the intestine. In the present study, we compared the adhesive properties of *E. coli* strains isolated from the colon of patients with IBD, cancer and those of controls. Of the adhesion-encoding virulence factors included in our study, the type-1 fimbrial adhesion (*fimH*) gene was the most frequent (72.5, 75, and 82 %) in *E. coli* isolated from healthy, IBD and cancer patient, respectively. Although *fimH* gene was higher in cancerous strains, there was no significant difference in comparison with isolated of control samples. Also *fimH* plays an important role in connection with dose and ultimately the development of disease (36). In our study the prevalence of *papC* gene was common, which is in accordance with previous studies (33, 37), but in contrast to other (38). Previous studies of adherent *E. coli *in IBD have produced variable results. Probing of fecal bacteria for adherence genes has revealed no differentiation from controls (39). Whereas augmented frequencies of adherent *E. coli* isolates have been found in ulcerative colitis and Crohn’s disease (40, 41). It seems likely that some of these discrepancies may have resulted from differences in bacterial isolation methods. Fluorescent on-site hybridization studies have shown that *E. coli* tend to be buried deep within the mucus layer in Crohn’s disease. Although close apposition of *E. coli* resulted in adherence gene by bacteria was not significant to this study, due to the high presence of these genes in the strains isolated from CRC and IBD, it is expected that this study could be consistent with other studies (8). This indicates that the increase in adhesion genes is associated with increased bacterial binding to the intestinal mucosa and eventually an increase in the incidence of intestinal malignancies. The high prevalence of several iron uptake genes, such as *ireA*, *fyuA*, and *iutA*, reflects their common occurrence in all *E. coli* strains. Like our study, the high prevalence of iron absorption genes was reported in isolated strains of the intestine (42). Far above the ground rate of *ireA* gene in groups IBD and cancer and in proportion to control, it shows the specific role of this gene in colonization. In general, for all virulence factors among four *E. coli* phylogroups, the prevalence of these virulence factors was highest in the phylogroups A and B2. These studies point out that bacterial biofilm are shaped in the mammalian large bowel and are related with and reliant on the mucus that lines the epithelium of the bowel (43, 44). Studies pointing at the importance of biofilm in the mammalian gut derive from a number of fields. And others working in parallel on factors that direct establishment and preservation of a spatially diversified gut microflora postulated that biofilm be supposed to be found as a part of the normal gut flora (45) dissimilar some other studies that have proven biofilm and CRC (46). In our study, the biofilm formation results for *E. -coli* strains isolated from cancer and inflammation were not significant in comparison with control subjects, which can be confirmed by the previous results that the formation of biofilm for normal flora of the intestine is a natural phenomenon. For this reason, biofilm formation as weak, moderate and strong forms is higher in isolated strains of normal individuals. There were no acceptable results in the study on the presence of the type of adhesion genes and its relationship with biofilm formation. Because reasonably few investigations have been voted out on microbial biofilm in the human digestive tract, little is really recognized about the formation and function of these entities, and with the possible exception of their interactions with the innate immune system, their metabolic and neuropathological significance to the host. The increasing shift in emphasis away from culture-based studies, and further development of molecular techniques (47), together with the emergence of methodologies for investigating gene expression in situ (48), will greatly facilitate future work on biofilm structures in the human colorectal. We experiential that *E. coli* were not limited to the tumor site of carcinoma as previously reported (8, 31).

In analyzes of phylogroups, adhesion and iron uptake factors and biofilms, the prevalence of B2 and A of phylogroups was higher in *E. coli* strains were isolated from people with CRC and IBD. The distribution of virulence factors in the two mentioned phylogroups was higher. The distribution of connecting factors among all three groups was close to each other and there was no significant difference between them. However, in people with cancer and inflammation, the frequency was slightly higher. The *ireA* gene had a significant difference (0.004) among the genes involved in iron acquisition. Our results showed the highest biofilm formation by *E. coli* isolated from normal individuals, which we concluded that biofilm formation could be the natural processes of *E. coli*, the normal flora of the intestine.
